# Matching expert range maps with species distribution model predictions

**DOI:** 10.1111/cobi.13492

**Published:** 2020-08-23

**Authors:** Kumar Mainali, Trevor Hefley, Leslie Ries, William F. Fagan

**Affiliations:** ^1^ Department of Biology University of Maryland 1210 Biology‐Psychology Building College Park MD 20742 U.S.A.; ^2^ Department of Statistics Kansas State University 205 Dickens Hall, 1116 Mid‐Campus Drive North Manhattan KS 66506 U.S.A.; ^3^ Department of Biology Georgetown University Reiss Science Building, Room 406, 37th and O Streets Washington DC NW 20057 U.S.A.

**Keywords:** concavity, detailed edge, distribution models, expert agreement, expert score, Glassberg, inhomogeneous Poisson point process, map porosity, Scott, species, acuerdo entre expertos, borde detallado, concavidad, Glassberg, modelos de distribución de especies, proceso de punto de Poisson no homogéneo, porosidad de mapa, puntaje de expertos, Scott, 专家评分, 专家协议, Glassberg 地图, Scott 地图, 物种分布模型, 非均匀泊松点过程, 函数凹性, 精细边缘, 地图孔隙度

## Abstract

Species’ range maps based on expert opinion are a critical resource for conservation planning. Expert maps are usually accompanied by species descriptions that specify sources of internal range heterogeneity, such as habitat associations, but these are rarely considered when using expert maps for analyses. We developed a quantitative metric (expert score) to evaluate the agreement between an expert map and a habitat probability surface obtained from a species distribution model. This method rewards both the avoidance of unsuitable sites and the inclusion of suitable sites in the expert map. We obtained expert maps of 330 butterfly species from each of 2 widely used North American sources (Glassberg [1999, 2001] and Scott [1986]) and computed species‐wise expert scores for each. Overall, the Glassberg maps secured higher expert scores than Scott (0.61 and 0.41, respectively) due to the specific rules (e.g., Glassberg only included regions where the species was known to reproduce whereas Scott included all areas a species expanded to each year) they used to include or exclude areas from ranges. The predictive performance of expert maps was almost always hampered by the inclusion of unsuitable sites, rather than by exclusion of suitable sites (deviance outside of expert maps was extremely low). Map topology was the primary predictor of expert performance rather than any factor related to species characteristics such as mobility. Given the heterogeneity and discontinuity of suitable landscapes, expert maps drawn with more detail are more likely to agree with species distribution models and thus minimize both commission and omission errors.

## Abstract 2

*Article impact statement*: Detailed expert maps are more likely to agree with species distribution models and thus minimize commission and omission errors.

## Abstract (es)

Concordancia entre los Mapas de Extensión Realizados por Expertos y las Predicciones de los Modelos de Distribución de Especies

**Resumen**

Los mapas de extensión de especies basados en la opinión de expertos son un recurso de suma importancia para la planeación de la conservación. Los mapas realizados por expertos generalmente van acompañados de las descripciones de las especies que detallan el origen de la heterogeneidad interna de la distribución, como las asociaciones entre hábitats, pero rara vez se consideran estas descripciones cuando se usan los mapas de expertos para un análisis. Desarrollamos una medida cuantitativa (puntaje de expertos) para evaluar la concordancia entre un mapa realizado por expertos y una superficie probable de hábitat obtenida a partir del modelo de distribución de especies (SDM). Este método recompensa tanto a la evasión de sitios inadecuados como a la inclusión de sitios adecuados en el mapa realizado por expertos. Obtuvimos los mapas realizados por expertos para 330 especies de mariposas a partir de dos fuentes norteamericanas usadas ampliamente (Glassberg [1999, 2001] y Scott [1986]) y calculamos los puntajes de expertos, hablando de cada especie, para cada mapa. En general, los mapas de Glassberg aseguraron puntajes de expertos más altos que los de Scott (0.61 y 0.41 respectivamente) debido a las reglas específicas (p. ej., Glassberg sólo incluyó las regiones en donde es sabido que la especie se reproduce, mientras que Scott incluyó todas las áreas a las que la especie se expandió cada año) que cada una usa para incluir o excluir áreas de las distribuciones. El desempeño pronosticado de los mapas realizados por expertos casi siempre se vio afectado por la inclusión de los sitios inadecuados, en lugar de estar afectado por la exclusión de sitios adecuados (la desviación fuera de los mapas realizados por expertos fue extremadamente baja). La topología del mapa fue el indicador primario del desempeño de los expertos en lugar de cualquier factor relacionado con las características de la especie, como la movilidad. Dada la heterogeneidad y la discontinuidad de los paisajes adecuados, los mapas realizados por expertos dibujados con mayor detalle tienen una mayor probabilidad de concordar con los SMD y por lo tanto minimizar los errores de comisión y de omisión.

## 【摘要】

基于专家意见的物种分布图是保护规划的重要资源。专家地图通常伴有对物种的描述, 详细说明了分布区内部的异质性来源, 如与栖息地有关的因素, 但在使用专家地图进行分析时却很少考虑这些信息。我们设计了一个定量度量指标 (专家评分) 来评估专家地图与从物种分布模型得到的栖息地概率图之间的一致性。这种方法看重专家地图中对不合适位点的避免以及对合适位点的纳入情况。我们从北美两个广泛使用的数据源 (Glassberg [1999, 2001]及Scott [1986]) 分别获得了330 种蝴蝶的专家地图, 并计算了每种蝴蝶的专家评分。总的来说, 由于用来包含或排除物种分布范围的特定规则存在差异 (如 Glassberg 地图只包含物种已知的繁殖区而 Scott 地图包括物种每年活动的所有地区), Glassberg 地图获得专家评分高于 Scott 地图 (分别为0.61和0.41) 。专家地图在预测中的问题更多在于包含了不合适的位点, 而不是排除了合适的位点 (专家地图之外的偏差非常低) 。专家地图表现情况的主要预测因素是地图的拓扑结构, 而非与物种特征相关的其它因素, 如移动性等。考虑到合适景观的异质性和间断性, 绘制得更为详细的专家地图更有可能与物种分布模型一致, 从而最大限度地减少错分误差和漏分误差。**【翻译: 胡怡思; 审校: 聂永刚】**

## Introduction

Data on species’ distributions are critical to conservation planning, predicting responses to climate change and public health (Parmesan 1996, 2006; Dawson et al. 2011; Mainali et al. 2015; Merow et al. 2017). Historically, a key source of such distributional data has been expert‐drawn range maps, which set boundaries on species’ likely occurrences.  Expert maps, which are developed for individual species based on a combination of distribution data and the collected experience and knowledge of naturalists, scholars, and others, delineate the geographical region in which a species is believed to occur (Hurlburt & Jetz 2007; Merow et al. 2017). As such, expert maps, which are available for thousands of species across diverse taxa, predict the binary state of species distributions as occupied or unoccupied, usually with a fairly coarse grain.

The utility of these maps ultimately depends on accuracy, but defining *accuracy* is difficult because the true distribution of a species cannot be known. When drawing maps, experts generally delineate a single region that includes the entire species range. This is done because any occupied areas falling outside of the delineated range clearly diminish map credibility (Hurlbert & White 2005; Hurlbert & Jetz 2007; Merow et al. 2017). Because of this emphasis on avoiding omission errors (false negatives), expert maps appear particularly good for delineating range edges beyond which a species is unlikely to occur (Jetz et al. 2012; Domisch et al. 2016). For birds, the boundaries of expert maps were reasonably accurate at 100–200 km resolution (Hurlbert & White 2005; Hurlbert & Jetz 2007; Merow et al. 2017), but predicted many false presences at finer resolutions (Hurlbert & White 2005; Hurlbert & Jetz 2007).

Expert maps are often used to identify biodiversity hotspots for conservation (Hurlbert & Jetz 2007) or estimate species richness (Hurlbert & White 2005). In such cases, multiple expert maps are stacked to obtain multispecies measures. Yet, expert maps have traditionally been developed specifically to accompany individual species accounts. Indeed, most expert‐drawn maps are supplemented by written species accounts that include ecological trait data such as habitat or elevational associations. This information, which is not included in the maps, makes it possible for readers to infer the internal heterogeneity of plotted species ranges.

Species distribution models (SDMs) provide an alternative to expert maps. Typically, SDMs are based on occurrence data and then interpolated and extrapolated to account for areas in which species may not have been seen, but are likely to occur due to a combination of environmental variables that correlate with known occurrences (Guisan & Thuiller 2005; Elith & Leathwick 2009). An SDM usually features fine spatial resolution and therefore captures fine‐grained heterogeneity of species ranges in ways that expert maps rarely do. Recent efforts leverage both expert maps and point data to improve SDMs (Domisch et al. 2016; Merow et al. 2017).

Because much conservation activity hinges on species ranges, metrics must be developed for assessing the accuracy of maps, whether expert‐drawn or implemented from statistical models. One way to judge the accuracy of expert maps is to compare them with each other, but we are not aware of any researchers who have done this. One challenge to this approach is that with no independent reason to prefer one over the other it becomes difficult to ascertain what conclusions to draw about the relative value of competing maps. A neutral benchmark provides a means of determining map quality. Although SDMs have several shortcomings (Kramer‐Schadt et al. 2013; Yackulic et al. 2013; Gomes et al. 2018), they are transparent, repeatable, and optimizable for particular purposes (e.g., by adjusting weights on commission vs. omission errors). We developed a metric that compares competing expert maps against carefully trained SDMs. Although we used SDMs as a benchmark for comparison, we do not suggest they represent truth. Instead, we devised an analytical way to use these SDMs as a neutral arbiter to evaluate range information when multiple expert‐drawn maps are available and a framework for understanding and judging map value. We explored this analytical framework with North‐American butterfly maps.

## Methods

### Map Development

We obtained digitized expert ranges of American butterfly species from 2 sources. James Scott (1986) published range maps after studying butterflies for 25 years. He reviewed several hundred references and consulted with over 100 experts. In drawing these ranges, Scott reported summer (where adult butterflies may be seen, even if they do not reproduce) and winter ranges (where the species are known to overwinter). The union of a species’ summer and winter ranges constituted its expert range here. We obtained digitized ranges for 541 species from Scott (1986) via the Map of Life project. Hereafter, this set of expert maps is referred to as Scott.

The second expert source comprised maps originally published in two books by Jeffrey Glassberg (1999, 2001) and subsequently updated by Glassberg. Based on published and unpublished literature, Glassberg created draft ranges by including places where butterflies regularly fly and produce at least 1 brood before dying back. Glassberg mapped the regions with 1, 2, or >2 broods. Thus, unlike Scott, if a species expanded its summer range but did not reproduce in those areas, those were not included in the range map, but rather denoted as strays and excluded from our analysis. The union of all these brood regions constituted the species range and represented areas where a species was known to reproduce, even if it did not overwinter. These draft ranges were then reviewed by 85 experts before Glassberg finalized the ranges. In 2014, we obtained digitized expert ranges of 659 butterfly species from this source, hereafter, referred to as Glassberg.

To provide a baseline for our analyses of expert maps, we sought occurrence records for American butterfly species and located 478,200 occurrence records from the North‐American Butterfly Association, 46,904 records from Butterflies and Moths of North America, and 137,431 records from Global Biodiversity Information Facility. To define the study area for each species, we first created an alpha hull (*α* = 8°), a generalization of the convex hull (Burgman & Fox 2003), around a species’ occurrence records to eliminate highly distant, isolated records. Second, we created a convex hull representing the spatial union of the alpha hull based on species occurrences and the two expert maps for that species. Third, we clipped the resulting convex hull by the boundary of land mass of the contiguous United States to obtain the study area (denoted *S*) of the species. Defined in this way, the study area for a species represented the spatial union of all relevant data sets. We developed SDMs for each butterfly species with the inhomogeneous Poisson point process (IPP) distribution approach (e.g., Warton & Shepherd 2010). Ultimately, we analyzed ranges for 330 species, after excluding species based on how much of their range was included in our main study area or because of conflicting taxonomy. We also ran the analysis separately for species whose ranges were 100% within our primary study area. See Supporting Information for details and rationale for SDM method and which species were included in analyses.

### Measuring Agreement Among Maps

We assumed each expert map was generated from a binary process, such that, within any subregion of the study area, the species were either present (y=1) or absent (y=0). To determine how close the expert maps agreed with the SDM predictions, we relied on the notion of a scoring rule for evaluating the accuracy of predictions of binary events (Table 1 in Gneiting & Raftery 2007). Because the IPP implies a Bernoulli distribution for occurrences, we used a proper scoring rule to appropriately match our SDM with the procedures used to evaluate the predictive accuracy of the expert maps (Cressie & Wikle 2015; Gneiting & Raferty 2007; Hefley & Hooten 2016). For a scoring rule, we used

(1) $$l\left(y_{j},p_{j}\right)=-2\log\left[{p_{j}}^{y_{j}}{\left(1-p_{j}\right)}^{1-y_{j}}\right],$$

where $y_{j}$ is equal to 1 if a map indicates that the *j*th small geographic area $A_{j}$ is occupied and 0 if the map indicates $A_{j}$ is unoccupied, and $p_{j}$ is the probability of occurrence in $A_{j}$. For a given species, the overall score for the study area is calculated by summing each $l(y_{j},p_{j})$ across *J* nonoverlapping $A_{j}$’s that fully partition the study area *S*. The expert's score is calculated as $\sum_{j\;=\;1}^{J}l\left(y_{j},p_{j}\right)$ for the set $S\;=\cup_{j=1}^{J}\;A_{j}$.

**Table 1:** Potential predictors of expert score, a quantitative metric developed to evaluate the agreement between an expert map and a habitat probability surface obtained from a species distribution model, and predictors of expert agreement, the fraction of the union of 2 expert maps that is common

Predictor and measure	Definition and level of categorical variable
Attributes of expert opinion maps^a^	
Polsby‐Popper index (range 0 to 1)	Ratio of the area of the map to the area of a circle whose circumference is equal to the perimeter of the map (Cox 1927)
Convex hull score (range 0 to 1)	Ratio of the area of a map to the area of the minimum convex polygon that encloses the map
Detailed edge (range 0 to ∞)	Ratio of the area of a map to its edge length
Attributes of occurrence‐based maps	
Number of occurrence points	Total number of occurrence records in the study area (as defined in Methods)
Moran's *I* (range –1 to 1)	Spatial heterogeneity of occurrences in the study area; score of the map is 1 for perfect clustering of similar values, 0 for perfect randomness, and –1 for perfect clustering of dissimilar values
Average density of occurrence points (count/10,000 km^2^)	
Butterfly life‐history traits^b^	
Mobility	Local, migratory, or mass migration
Habitat breadth	Generalist: associated with many specific ecotypes (e.g., fields, meadows, prairies, and pastures), although they may have particular canopy requirements (e.g., no canopy rather than closed forest); specialist: associated with specific ecotypes (e.g., tall‐grass prairies); narrow: narrow but not specialized habitat associations
Host plant	One genus of plants; few (≤5) species of a plant family; many species of a plant family; few species of several plant families; many species of several plant families; dead plant tissue
Taxonomic family	
Oviposition	Single, cluster, or both
Overwintering state	Egg, larvae, pupa, or adult
Voltinism	Univoltine (obligate 1 flight/year), bivoltine (obligate 2 flights/year), or multivoltine (1 to many flights depending on length of season)
Local abundance	Common, uncommon, or irruptive
Distribution	Local: generally found in localized sites; widespread: could be found anywhere within the canopy, habitat, and range of the species; stray: not known to breed in the area or be a regular migrant to the area; individuals seen only sporadically
Average wing span	
Wing span range	Maximum wingspan minus minimum wingspan

^a^Perimeter and area of map measured on an ellipsoid representation of Earth in kilometers and square kilometers, respectively.

^b^All predictors categorical except Average wing span.

Other types of scoring rules could be used instead of Eq. 1. For example, area under the curve (AUC) is widely used in ecology, but this scoring rule is not proper (i.e., it is possible to find better AUC scores when the estimated probabilities of occurrence differ from the true probabilities of occurrence) (Byrne 2016). Proper scoring rules like Eq. 1 ensure that the best value of the scoring rule is achieved when the estimated probabilities of occurrence match the true values (Byrne 2016).

We used the results of the SDM as an independent arbiter of truth without assuming that it necessarily outperforms either expert map. We restricted our models to regions in which occurrence is highly probable, and this greatly lessened the typical SDM problem of finding the true area of occurrence.

Although useful, the score produced by Eq. 1 depends on how the $A_{j}$s are chosen to partition the study area *S*. That is, the map's score, $\sum_{j\;=\;1}^{J}l\left(y_{j},p_{j}\right)$, depends on grid resolution, which is arbitrary. We set the grid resolution at 10 arc minutes. Because the SDM provides predictions in continuous geographic space, we needed to define the continuous analog of the occupancy probability as

(2) $$p\left(s\right)=\lim_{|A|\to 0}1-e^{-\bar{\lambda }},$$

where $\bar{\lambda }=\int_{A}\lambda \left(s\right)ds$, *s* is a vector of coordinates and λ(s) is an IPP estimated intensity function the log of which is specified as a linear combination of location‐specific covariates of species distribution (Supporting Information).

Likewise, a map is a binary process that exists in continuous geographic space, such that at any point, the map indicates that the species is present, y(s)=1, or absent, y(s)=0. With spatially continuous specifications of y(s) and p(s), the map's score is calculated as

(3) $$E\;\left\{l\left[y(s),p(s)\right]\right\}=\int_{S\;}\;l\left[y(s),p(s)\right]ds,$$

which we refer to as the deviance. This deviance measures how close each expert's map is to the intensity function from the SDM built from the occurrence records for that species. However, because this measure in Eq. 3 is defined in continuous geographic space, it is no longer sensitive to an arbitrary choice of how the study area *S* is partitioned. In practice, the integral in Eq. 3 is evaluated using a numerical quadrature approximation (Givens & Hoeting 2012). Furthermore, p(s) is a function of λ(s), which must be estimated from the occurrence records. Thus, the deviance is estimated by plugging in the estimated value $\hat{\lambda }\left(s\right)$ in place of λ(s).

**Figure 1:**
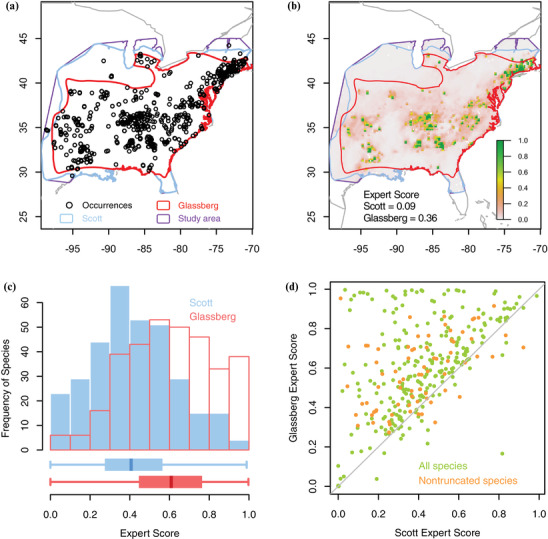
Butterfly distributional data and expert score (Eq. 4) reflecting the performance of 2 sources of experts’ range maps compared with the (continuous) probability of occupancy, p(s) (value close to 1, strong agreement between expert range and species distribution modeling predictions; value close to 0, no ability to differentiate suitable landscape from unsuitable). For *Achalarus lyciades* (a) expert range maps from Scott and Glassberg and occurrence records from North American Butterfly Association, Butterflies and Moths of North America, and Global Biodiversity Information Facility and (b) predicted habitat suitability based on species distribution models relative to expert maps (legend shows habitat suitability range) (1, high quality habitat). (c) Scott and Glassberg expert scores for 330 species in the study. (d) Glassberg expert score relative to Scott expert score for all 330 species.

Because the deviance in Eq. 3 is a relative measure, we needed a null map to characterize the predictive value of expert maps. We defined the null map for a species as that which covers the entire study area *S* (Fig. 1a). We propose a deviance‐explained metric as

(4) $$\mathrm{expert}\;\mathrm{score}\;=\;1-\;\frac{E\left\{l\left[y(s),p(s)\right]\right\}}{E\left\{l\left[z(s),p(s)\right]\right\}},$$

where E{l[y(s),p(s)]} is the deviance given the expert's map (i.e., y(s)) and E{l[z(s),p(s)]} is the deviance given the null map (i.e., z(s)). The expert score has an interpretation similar to the familiar coefficient of determination from simple linear regression or the more general pseudocoefficient of determination for generalized linear models. For example, when the expert score equals 0, the expert map has predictive accuracy equal to that of the null map. A higher expert score indicates a closer match of expert map with highly suitable sites. Expert score can be negative when an expert map has less predictive accuracy than the null map.

The deviance in Eq. 3 can be decomposed to represent the contribution to the deviance score due to omission and commission errors. More specifically, for any expert map, the study area *S* can be partitioned into two disjointed sets, S=I∪O, where *I* is the area inside the expert's map and *O* is the area outside the expert's map but within *S*. With this partition, the integral in Eq. 3 is

(5) $$\begin{array}{ccc} E\;\left\{l\left[y(s),p(s)\right]\right\} & = & \int_{I\;}\;l\left[y(s),p(s)\right]ds\\ & & +\int_{O}l\left[y(s),p(s)\right]ds.\; \end{array}$$

The first term on the right side of Eq. 5 is the deviance inside the expert map, which, when scaled by the null deviance, quantifies commission error (error of predicting unsuitable landscape as part the expert map). Similarly, the second term on the right side in Eq. 5 is the deviance outside the expert map, which, when scaled by the null deviance, quantifies omission error (error of predicting suitable landscape outside the expert map). Thus, this decomposition in Eq. 5 results in a natural interpretation of the expert score as [1 – (scaled deviance inside + scaled deviance outside)].

Glassberg and Scott used different ecological phenomenon to define their ranges. Therefore, we did not expect them to match exactly even if they both could be assumed to represent truth as they defined it. Nevertheless, we believed it was useful to directly compare the overlap in the two maps, especially because expert maps are often used without regard to the specific rules used to generate them. For each species, we quantified agreement between the two expert maps as:

(6) $$\mathrm{expert}\;\mathrm{agreement}\;=\;\frac{\mathrm{area}\;\mathrm{of}\;\mathrm{the}\;\mathrm{intersection}}{\mathrm{area}\;\mathrm{of}\;\mathrm{the}\;\mathrm{union}}$$

This measure scales from 0 (complete disagreement between corresponding expert maps) to 1 (complete agreement). Next, we used three groups of explanatory variables to predict expert score (defined in Eq. 4) in each of the 2 sources of expert maps and to predict expert agreement between 2 expert maps. Specifically, we considered expert map geometry (3 predictors), occurrence records geometry (3 predictors), and life‐history and ecological traits of butterfly species (11 predictors) (details in Table 1). Predictors related to expert map attributes and to occurrence records were calculated directly from the expert maps and the occurrence data, respectively. Predictors related to life‐history and ecological traits were compiled from Scott (1986), Opler and Malikul (1992), Bird (1995), Glassberg (1999), Opler (1999), Daniels (2003), and Bouseman et al. (2006).

We used multimodel inference (Anderson & Burnham 2004; Burnham & Anderson 2004) to quantify the utility of these three groups of variables as significant predictors of expert score and, separately, of expert agreement. For each of the 3 groups of predictors, we exhaustively examined the performance of all possible main effects models built from the combinations of predictors in that group. For example, we had 7 main effects models for expert map geometry (three models with individual predictors plus three bivariate models plus a model with all three predictors). Each of these models was assigned a probability based on the Akaike Information Criterion (AICc) corrected for small sample sizes, such that the summed probability of the models was 1 (Buckland et al. 1997; Calcagno & de Mazancourt 2010). A predictor was deemed important if the sum of the probability of all models that included that parameter was ≥0.8 (Calcagno & de Mazancourt 2010). We then created a linear model with the important predictors from the map geometry group.

We repeated the method for the groups of predictors related to occurrence records (7 models) and life‐history and ecological traits (2047 models) and identified important predictors in each of these groups. Eventually, we created models with important predictors from more than one group. For each of these models, we report goodness of fit as adjusted *R* ^2^.

Data analyses and plotting were performed in R × 64 3.5.1 (R Project for Statistical Computing) with the following libraries: gbm (for the main analysis of SDM), raster, maptools, maps, rgdal, geosphere, rgeos, scales, alphahull, sp, rgbif, plyr, mandeR, spatialEco, Hmisc, glmulti, magrittr, DT, htmlwidgets, ggplot2, officer, flextable, jtools, venneuler, and cvAUC. We developed an R package called expertscore for computing the metric we developed; the library can be downloaded from https://github.com/kpmainali/expertscore.

## Results

### Distributional Data and Species Distribution Models

Spatial overlap between the two sets of expert maps and between each set of expert maps and the occurrence records varied by species (Fig. 1a). Modeling the distribution of a species based on its occurrence records and environmental covariates yielded a probability surface for the occurrence of that species in its specific study area (Fig. 1b illustrates this process for one species). Usually, these surfaces included very low probabilities toward the edges of the study area, but exceptions occurred when land and water boundaries truncated the range or when occurrence records were clustered near an artificial (geopolitical) truncation boundary. Species distribution models, when evaluated by AUC score, performed very well. Following the rule of thumb that an AUC score of 0.8–0.9 implies a good model and >0.9 implies an excellent model (Araújo et al. 2005), >98% of species scored excellent and the rest scored good.

### Expert Scores and Predictors

**Figure 2:**
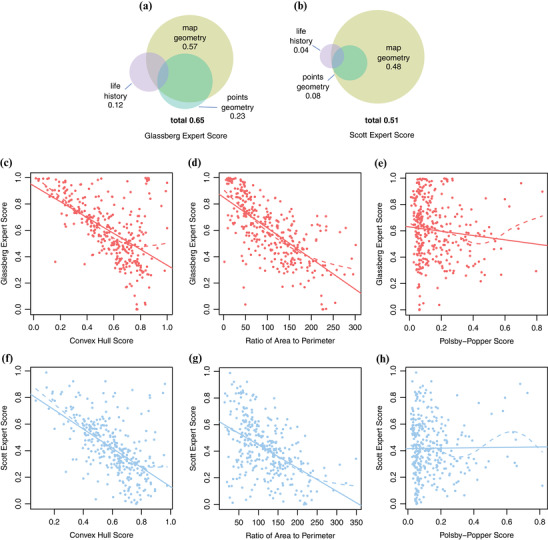
Predictors of expert score of species range maps of Glassberg and Scott. Score predicted separately by the covariates related to map geometry, occurrence geometry, and life history. Groups of covariates were merged in all combinations to predict (a) Glassberg expert scores and (b) Scott expert scores (circle size, relative measure of variance explained by each group, adjusted *R* ^2^ shown; circle overlap, measure of how much of the explanatory power of a group of covariates is absorbed by another group when multiple groups are included in the model; total, variance explained by all 3 groups collectively). Significant predictors related to map geometry for the (c‐e) Glassberg score and (f‐h) Scott score.

When considering all species, expert score for Glassberg was substantially higher than for Scott (median = 0.61 vs. 0.41, respectively) (Fig. 1c). On a pairwise basis, Glassberg Expert Score exceeded Scott Expert Score for 86% of species (Fig. 1d). Both coefficients decreased with increasing expert range size (Supporting Information). Several predictors related to attributes of map geometry and attributes of occurrence records, and butterfly traits were significant predictors of expert score for both the Glassberg and Scott data sets (Fig. 2a,b & Table 2). Attributes of the maps themselves constituted the strongest predictors of expert score (adjusted *R* ^2^ = 0.57 for Glassberg and 0.48 for Scott). Glassberg and Scott maps were also similar in that predictors related to occurrence records and to species ecology or life‐history traits explained much less variance in expert score than attributes of map geometry and in that the variance explained by these 2 groups of predictors added little to models that already included attributes of map geometry (Fig. 2a,b & Table 2). Hence, compared with models built only from predictors of map geometry, models also including predictors based on occurrence and ecology and life history improved only slightly (adjusted *R* ^2^: 0.57 vs. 0.65 for Glassberg and 0.48 vs. 0.51 for Scott). For both sets of expert maps, the convex hull score and detailed edge score were more strongly related to expert score than was the Polsby‐Popper index (Fig. 2c‐h) (definitions in Table 1).

**Table 2:** Significant predictors of expert score and expert agreement.^*^

	Dependent variable					
	Glassberg expert score		Scott expert score		Glassberg–Scott agreement	
Predictors	All species	Nontruncated species	All species	Nontruncated species	All species	Nontruncated species
Attributes of expert opinion maps						
Polsby‐Popper index	Yes	Yes	Yes	Yes	Excluded	Excluded
Convex hull score	Yes	Yes	Yes	Yes	Excluded	Excluded
Detailed edge	Yes	Yes	Yes		Excluded	Excluded
Adjusted *R* ^2^	0.57	0.57	0.48	0.44		
Attributes of occurrence‐based maps						
Number of occurrence points	Yes	Yes	Yes		Yes	Yes
Moran's *I*	Yes	Yes			Yes	Yes
Average density of occurrence points					Yes	Yes
Adjusted *R* ^2^	0.23	0.20	0.08	0	0.30	0.45
Life‐history traits of butterfly						
Mobility	Yes					
Habitat breadth						Yes
Host plant use					Yes	
Taxonomic family					Yes	
Oviposition						
Overwintering state					Yes	Yes
Voltinism	Yes		Yes		Yes	
Local abundance						
Distribution						
Average wing span						
Wing span range						
Adjusted *r* ^2^	0.12	0	0.04	0	0.29	0.58

^*^ *Yes* indicates significant predictors of each of the dependent variables. Significant predictors identified separately for each of the following 3 groups of predictors: attributes of expert opinion maps, attributes of occurrence‐based maps, and life‐history traits of the butterfly. The explained variance is reported separately for the 3 groups of predictors (also shown in Fig. 2a,b). Overall model fits, which draw on all groups of predictors, are in Fig. 2a,b. *excluded* indicates predictors excluded by the structure of particular analyses. Attributes of Glassberg map are excluded as predictors of Scott map and vice versa.

Results were broadly similar when we considered only those species with nontruncated ranges (Supporting Information). Standardized coefficients of significant predictors are in Supporting Information. The degree to which expert score was predicted by each of the map geometries under the simple linear regression framework is summarized in Supporting Information.

### Expert Agreement and Predictors

**Figure 3:**
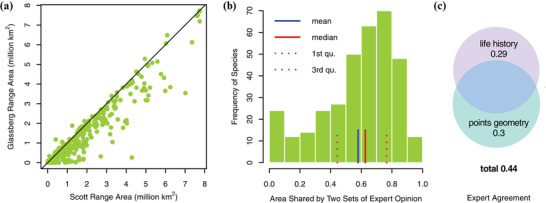
Expert agreement between Glassberg and Scott and predictors of expert agreement: (a) area of Scott range versus Glassberg range (line, 1:1), (b) expert agreement between Glassberg and Scott, and (c) expert agreement predicted separately based on covariates related to occurrence geometry and life history (2 groups of covariates merged to predict expert agreement; circle interpretation as in Fig 2). Bivariate plots of expert agreement with each of the significant predictors related to points geometry and to ecology and life history are in Supporting Information.

Across all species, Scott range size was consistently greater than the corresponding Glassberg range size (for 87% of species) (Fig. 3a). Expert Agreement ranged between almost complete agreement to complete disagreement (Fig. 3b). Across all species, the 2 expert maps shared an average of 58% of the total range (median = 62%). Expert Agreement increased monotonically as a function of range area for each of the sets of expert maps (not shown). However, we excluded expert range area and other map attributes as predictors of expert agreement to avoid issues of circularity. Predictors related to attributes of occurrences and ecology and life history explained 30% and 29% of the variance in expert agreement, respectively (Fig. 3c & Table 2); collectively, they explained 44% of the variance. Scott and Glassberg expert maps agreed more for those species represented by a larger number of occurrence records but agreed less for species whose occurrences featured greater heterogeneity in spatial distribution (Supporting Information). Point density exhibited a weak negative relationship with expert agreement, and several ecological and life‐history traits were also weakly associated with expert agreement (Supporting Information).

Restricting these same analyses to those species with nontruncated ranges, we found 45% of variance among maps explained by attributes of occurrences (vs. 30% for all species), 58% explained by ecology and life‐history traits (vs. 29% for all species), and 64% explained by both groups of predictors (vs. to 44% for all species) (Supporting Information).

### Deviance in Expert Maps

**Figure 4:**
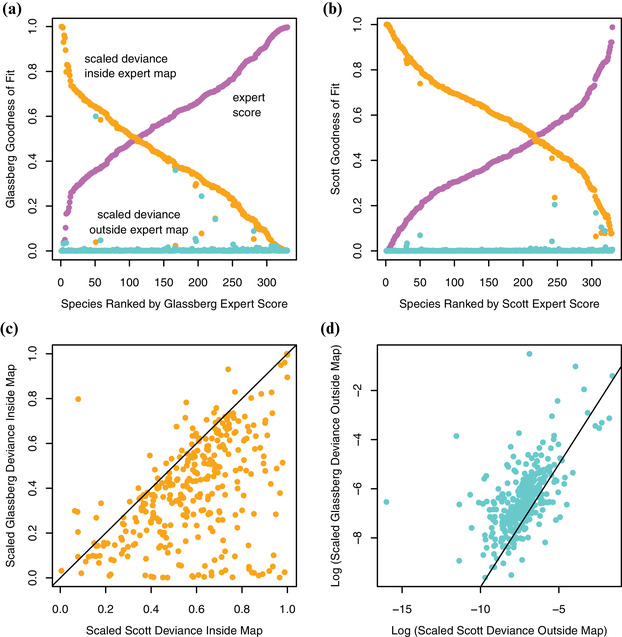
Decomposing expert scores into deviance inside and outside expert maps for (a) Glassberg and (b) Scott. The deviance of the expert map as a fraction of null deviance inside the map (scaled deviance inside expert map) and outside of map (scaled deviance outside expert map) should be low for high expert scores. (c) Substantial differences inside the maps and (d) trivial differences outside the maps between Glassberg and Scott prediction deviance.

Expert maps performed well in predicting unsuitable landscape outside the expert‐drawn map boundaries. This was true for both Glassberg and Scott maps and for the vast majority of species, as indicated by very low deviance outside scores irrespective of expert score (Fig. 4a,b). Consequently, the overall performance of the expert maps was almost exclusively determined by the deviance inside (i.e., from predictions concerning unsuitable areas inside the map).

Deviance inside was lower for Glassberg than for Scott for 88% of species (Fig. 4c). This indicated that Glassberg more robustly predicted suitable sites than Scott. In contrast, 80% of species had their deviance outside higher for Glassberg than for Scott (Fig. 4d), indicating the Glassberg maps had more omission errors than the Scott maps. However, omission error contributed little to overall model performance (Fig. 4a,b).

## Discussion

Expert opinions, especially opinions that represent the collective consensus of many experts, are indispensable components of knowledge. Such opinions are especially useful when more objective knowledge is incomplete, as is true for species distributions. Different experts draw maps with different intentions, which can result in surprising deviations from each other in terms of range boundaries. Only rarely are alternative expert maps available for comparison. We contrasted alternative expert maps for diverse species via comparisons to SDMs that value reducing errors of commission as well as omission.

Expert maps are often drawn specifically to reduce omission errors and deal with commission errors by having accompanying text describing where species are most likely to be seen within the drawn range boundaries. Consequently, expert range maps make substantial commission errors, often including large amounts of uninhabited land within the range boundaries. For example, species of birds, on average, occur in only 40% of surveyed sites (Hurlbert & White 2005) or about half of the 0.25° grid cells (Hurlbert & Jetz 2007). Our analyses of expert maps echo these findings when we treated the SDMs as the reference point (Fig. 4a,b). Our efforts feature two advances. First, we addressed errors arising from the inclusion of unsuitable sites in the expert maps as well as errors arising from the exclusion of highly suitable sites from the expert map. Second, rather than evaluating the expert maps against presence‐absence grids based on occurrence records (Hurlbert & White 2005; Hurlbert & Jetz 2007), we evaluated expert maps relative to gridded probability surfaces derived from SDMs. This alternative may be especially advantageous when, as here, highly efficient SDMs are available that can detect habitat and likely occupied habitats in unsampled locations.

For both the Scott and Glassberg expert map sets, we observed substantial interspecific differences in agreement with the SDMs (expert score, Fig. 1b). Thus, the same expert method can yield very different predictions relative to occurrence‐based models when applied across diverse species. Overall, the Glassberg maps provided better matches to the SDM maps for 86% of species (Fig. 1c), indicating a consistent consequence of the different approaches the 2 authors took when delineating expert maps. For instance, Glassberg only included regions in his range where the species was known to reproduce even if it does not overwinter, whereas Scott included areas that the species expanded to each year, even if it did not reproduce. Thus, not surprisingly, the Glassberg range was smaller than the corresponding Scott range for the vast majority of the species (Fig. 3a). This suggests the higher fraction of false positives that occurs in large expert ranges reduces the degree to which those maps will agree with the predictions of SDMs.

Indeed, by contrasting the performance of expert maps inside and outside expert‐drawn map boundaries, we found that for almost all species the predictive performance of the expert maps was penalized by inclusion of unsuitable sites within the map, rather than by exclusion of suitable sites outside of the map (Fig. 4a,b). This reflects the strategies used in drawing the expert maps: experts routinely delineate the boundaries of a species’ range and then allow their accompanying species description to provide guidance on internal heterogeneity. Overall, the two sets of expert maps differ greatly in the extent to which they include false positives inside the map boundaries. By including nonbreeding ranges, maps that are overly generous in space may have reduced utility (Fig. 1c) if they include unsuitable sites inside (Fig. 4c) because they stretch to include more strays or vagrants (Fig. 4d).

Expert maps vary in important ways. Among birds, which have some of the most complete distributional information (Hurlbert & Jetz 2007), expert maps are a reasonable approximation of species’ range at 100–200 km spatial scale (Hurlbert & White 2005; Hurlbert & Jetz 2007; Merow et al. 2017). For many other taxonomic groups, whose distributions are less well known than birds, the spatial accuracy of expert maps is largely unknown. Even for birds, expert maps tend to include false presences at coarse resolutions (Hurlbert & Jetz 2007), limiting the utility of the expert maps for understanding ecological processes, conservation planning, disease risk assessment, and similar applications unless the accompanying text is also taken into account. However, when used for analytical purposes, usually only the range map is considered. The framework we developed offers a way to explore the congruence between expert maps and SDMs, which are very different approaches for understanding species ranges. An emphasis in minimizing omission errors more strongly than commission errors shifts an SDM output from detailed edge and heterogeneous and disjointed patches of highly suitable areas to a smooth blob‐like area commonly reported in expert maps.

One caveat of using a probability surface to evaluate expert credibility is that SDM outputs cannot possibly account for all dispersal barriers, biotic interactions (Soberón 2007), and environmental dependencies. These omissions could result in overestimation of species distribution, whereas experts would be expected to know about such geographical and biological constraints (Domisch et al. 2016), thereby, yielding more realistic species range maps. If such omissions were important, they would drive deviance outside scores upward. However, we found near‐0 deviance outside (Fig. 4a,b). In contrast, SDMs may not capture range internal heterogeneity due to spatial biases in available data (Kramer‐Schadt et al. 2013; Yackulic et al. 2013). This important problem can be at least partially addressed if species occurrences are well sampled across multiple environmental gradients. Such sampling reduces bias along the variables used to predict occurrence patterns, even if the sampling is geographically biased. A comprehensive review of the best practices in SDM is beyond the scope of this article (see contemporary literature, including Elith & Leathwick 2009 and Araújo et al. 2019).

### Lessons for the use of Expert Maps

Organisms typically tolerate environmental conditions across a continuous range, with upper and lower critical limits on either side of an optimum (Miller & Stillman 2012). However, continuity in environmental ranges need not map onto continuity in geographic space. Consequently, mapped ranges may feature porosities reflecting unsuitable localities within a larger suitable region (Hurlbert & White 2005); tortuous range edges that result in range concavities and increased detailed edge measures; and disjunct suitable areas. Here, agreement between expert maps and SDMs decreased with increases in 3 expert map traits: convex hull score, detailed edge, and Polsby‐Popper score (Fig. 2d‐i) (definitions in Table 1). Collectively, these 3 measures characterize the geometric shape of the maps: were the maps elongate versus compact, convex or featuring concavities, and drawn with much or little boundary detail. Once these differences in range geometry were accounted for, life‐history and ecological traits explained almost no further variability, which was a surprising result (Fig. 2).

Because expert maps are generally presented with accompanying information about habitat, elevational, or other physical environmental preferences, actual internal heterogeneity is routinely absent from the maps. Increased accessibility to relevant environmental layers may allow future expert maps to be combined within geographic information systems to minimize both omission and commission errors. Effectively this would lead to expert maps that feature porosities or are split into separate spatial units to eliminate unsuitable landscape. None of the maps we used featured porosities, nor did they allow a reliable counting of separate spatial units.

Conservation practitioners are far more likely to use existing expert maps than to develop new ones. As we show, not all expert maps are drawn based on the same criteria; thus, expert maps are not always comparable (either for different species by the same group of experts or for the same species by different groups of experts). Practitioners must be especially careful to understand how expert maps were developed and what assumptions were presented by the authors. We found expert maps with small convex hull scores and small area to perimeter ratios were more likely to match predictions from species distribution models (Fig. 2). It remains to be seen if this is more generally true.

In conclusion, range maps are abstractions of experts’ belief about species distributions. Those abstractions depend on spatial scale and thus may introduce errors especially when they are digitized and stacked for uses beyond which they were intended such as estimating local species richness (Hurlbert & Jetz 2007). However, expert range maps are widely used in macroecological and conservation analyses (e.g., Shriner et al. 2006) precisely because they are so readily available. It is clear, however, that expert maps should be used with caution and with clear attention to the assumptions originally used to draw them. In recent analyses, expert range maps were used in 69% of 85 studies of species richness (Hawkins et al. 2003), even though they predicted roughly as many false occupancies as true occupancies (Hurlbert & White 2005; Hurlbert & Jetz 2007) and overestimated the spatial pattern of species richness (Hurlbert & Jetz 2007). Our analyses suggest that expert range maps will provide the best matches to SDMs when they are drawn to reduce errors of commission and when they feature heightened values of concavity and detailed edge. When available, we predict that other map characteristics, such as porosity and number of spatially isolated units, will also contribute to the agreement between SDMs and expert maps. Likewise, SDMs could leverage the value of expert maps (Jetz et al. 2012) if the maps are used to limit the boundaries of potential space (Fig. 1a), thus, helping to reduce SDM commission errors from the outset.

## Supporting information

Steps undertaken to achieve the final set of 330 species analyzes (Appendix S1), detailed methods for building species distribution models (SDMs) using inhomogeneous Poisson point processes (Appendix S2), a synopsis of the advantages of building SDMs in this way (Appendix S3), a plot of expert score against range area for both sets of expert maps (Appendix S4), a plot of explanatory variables of Expert Score for species with non‐truncated ranges (Appendix S5), a plot of significant predictors of Glassberg Expert Score (Appendix S6), a plot of significant predictors of Scott Expert Score (Appendix S7), plots of Expert Agreement against explanatory variables (Appendix S8), a plot of significant predictors of Expert Agreement (Appendix S9), a plot of variance in Expert Agreement for species with non‐truncated ranges (Appendix S10), and a table of simple linear regression models predicting expert score by map geometries (Appendix S11) are available online. The authors are solely responsible for the content and functionality of these materials. Queries (other than absence of the material) should be directed to the corresponding author.
