# Supplementary material for: Matching expert range maps with species distribution model predictions
Source: Conserv Biol. 2020 Aug 23;34(5):1292–304. doi: 10.1111/cobi.13492 (PMC7540670; doi:10.1111/cobi.13492)
Supplement: Supplementary file 1 — Steps undertaken to achieve the final set of 330 species analyzes (Appendix S1), detailed methods for building species distribution models (SDMs) using inhomogeneous Poisson point processes (Appendix S2), a synopsis of the advantages of building SDMs in this way (Appendix S3), a plot of expert score against range area for both sets of expert maps (Appendix S4), a plot of explanatory variables of Expert Score for species with non‐truncated ranges (Appendix S5), a plot of significant predictors of Glassberg Expert Score (Appendix S6), a plot of significant predictors of Scott Expert Score (Appendix S7), plots of Expert Agreement against explanatory variables (Appendix S8), a plot of significant predictors of Expert Agreement (Appendix S9), a plot of variance in Expert Agreement for species with non‐truncated ranges (Appendix S10), and a table of simple linear regression models predicting expert score by map geometries (Appendix S11) are available online. The authors are solely responsible for the content and functionality of these materials. Queries (other than absence of the material) should be directed to the corresponding author. [file COBI-34-1292-s001.docx]

**SUPPORTING INFORMATION**

**Appendix S1. Obtaining the final set of butterfly species for analysis**

Of all the maps common across the Scott and Glassberg datasets, we dropped 17 maps for irreconcilable differences in species taxonomy across sources (which caused the ranges to be not comparable), 42 maps because the maps included only subspecies, 93 maps for species with <30 occurrences, and 6 cases where the maps were not comparable across sources. In addition, to exclude species that were only marginally within the mapped regions, we instituted a range requirement. For this, we only included species where the majority of the disjunct winter range (as defined by Scott) occurred in the portion of North America treated by both sources, which included the contiguous United States and a southern zone of Canada. In addition, we only included species whose winter range or summer range (as defined by Scott) extended at least 100 or 250 miles, respectively, into the commonly mapped area. This was to avoid including species when the vast majority of their range was outside of our study region and we would only be comparing the very edge of their range. This range requirement excluded an additional 34 species. Finally, we retained a set of 330 species that had quality data from both sets of expert-drawn maps, as well as sufficient occurrence records to build an SDM.

Of the 330 species included in the analysis, the expert-drawn ranges extended outside the contiguous 48 United States for 256 species, whereas the remaining 74 species had their complete range inside the contiguous US. Glassberg reported ranges extending to both Canada and Mexico, whereas Scott reported ranges extending only to Canada. Because ranges with an artificial truncation at different locations cannot be compared, we truncated all ranges by the boundary of the contiguous US. We conducted separate analyses on the set of all 330 species and on the set of 74 species whose ranges were never truncated.

**Appendix S2. Building continuous species distribution models from discrete occurrence data**

Rather than subdividing spatial regions into grid cells that could be scored for presence/absence, we viewed species ranges as bounded within continuous geographic space. A species’ occurrence pattern within that space was determined by an inhomogeneous Poisson point process (IPP) distribution, which is a type of SDM (Warton & Shepherd 2010; Aarts et al. 2012; Renner & Warton 2013; Renner et al. 2015; Hefley & Hooten 2016). The IPP is governed by intensity function, $\lambda(\mathbf{s})$, where **s** is the coordinate vector of a single point contained within the study area $\mathcal{S}$. To build a species distribution model, this intensity function can be specified as a linear combination of location-specific covariates with

$log (\lambda(\mathbf{s})) = \beta_{0} + {\mathbf{x}(\mathbf{s})}^{\boldsymbol{'}} \boldsymbol{\beta}$, (1)

where $\beta_{0}$is the intercept, $\mathbf{x}(\mathbf{s})$ is a *p* × 1 vector that contains the covariates at location $\mathbf{s}$, and $\boldsymbol{\beta}\equiv(\beta_{1},\beta_{2},\ldots,\beta_{p})^{'}$is a vector of regression coefficients. For our analyses to build species distribution model, we considered 28 spatially dependent covariates to model butterfly distributions. These were growing degree days calculated from DayMet data (Thornton et al. 2014) with respect to a base temperature of 11.5°C (Zalucki 1982), land cover using the classification system of Wickham et al. (2014) and 26 bioclimatic variables obtained from the CliMond dataset (Kriticos et al. 2012). These covariates and their sources are reported in Supp Table 1.

Estimating the intensity function for a species required fitting the IPP distribution for that species to its occurrence records. We used boosted regression trees (BRTs) to estimate the intensity function of the IPP distribution (Fithian & Hastie 2013). IPP provides a flexible framework to model the intensity function, which represents the species distribution as a continuous surface. For example, a regression-type model such as Eq. 1 could be used if interpretation of the regression coefficients was an important goal of the study. Likewise, more flexible approaches that rely on regression trees (Mainali et al. 2015) could be used if, as in our study, maximizing predictive accuracy was the goal. Elith et al. (2008) provide a guide to BRTs for ecological data, and Fithian & Hastie (2013) demonstrate how to estimate the intensity function of the IPP distribution. The intensity function estimated onto a continuous probability surface is simply integrated over the area of interest and transformed to probability on a discrete geographic area (Cressie & Wikle 2015; Hefley & Hooten 2016).

References Cited:

Aarts G, Fieberg J, Matthiopoulos J. 2012. Comparative interpretation of count, presence--absence and point methods for species distribution models. Methods in Ecology and Evolution **3**:177–187.

Elith J, Leathwick JR, Hastie T. 2008. A working guide to boosted regression trees. Journal of Animal Ecology **77**:802–813.

Fithian W, Hastie T. 2013. Finite-sample equivalence in statistical models for presence-only data. The annals of applied statistics **7**:1917.

Kriticos DJ, Webber BL, Leriche A, Ota N, Macadam I, Bathols J, Scott JK. 2012. CliMond: global high-resolution historical and future scenario climate surfaces for bioclimatic modelling. Methods in Ecology and Evolution **3**:53–64.

Renner IW, Elith J, Baddeley A, Fithian W, Hastie T, Phillips SJ, Popovic G, Warton DI. 2015. Point process models for presence-only analysis. Methods in Ecology and Evolution **6**:366–379.

Renner IW, Warton DI. 2013. Equivalence of MAXENT and Poisson point process models for species distribution modeling in ecology. Biometrics **69**:274–281.

Thornton PE, Thornton MM, Mayer BW, Wilhelmi N, Wei Y, Devarakonda R, Cook RB. 2014. Daymet: Daily Surface Weather Data on a 1-km Grid for North America, Version 2.

Wickham J, Homer C, Vogelmann J, McKerrow A, Mueller R, Herold N, Coulston J. 2014. The multi-resolution land characteristics (MRLC) consortium—20 years of development and integration of USA national land cover data. Remote Sensing **6**:7424–7441.

Zalucki MP. 1982. Temperature and rate of development in Danaus plexippus L. and D. chrysippus L.(Lepidoptera: Nymphalidae). Australian Journal of Entomology **21**:241–246.

**Appendix S3**

**Why IPP models should be preferred over traditional SDM**

In addition to providing a framework that represents a species distribution as a continuous surface rather than a grid, using the IPP distribution for an SDM alleviates some of the technical issues associated with traditional techniques. For example, a common problem when using traditional SDM is defining the number and location of the so-called “pseudo-absence” points (e.g., Warton & Shepherd (2010)), which are user-defined locations that are selected and treated as data. The IPP provides insight into the role of the “pseudo-absence,” which are required to compute the likelihood via integral approximation methods (Warton & Shepherd 2010).

An important problem of traditional SDMs relates to the size of the geographic range from which the ‘background points’ are drawn. Increasing geographic background artificially inflates AUC (Lobo et al. 2008), but gives poor prediction of species distribution (Acevedo et al. 2012), because adding more background points to the sample changes the overall probability of occurrence, making the predictions meaningless (Warton & Shepherd 2010; Fithian & Hastie 2013). This issue of scale dependence in logistic regression models used in traditional SDM can be resolved by IPP which is scale-free (Renner et al. 2015). The IPP assumes a continuous environmental space resulting in an intensity function which is free of spatial resolution issues. The intensity function is transformed and then integrated which converts it into a probability of occurrence for a selected grid cell size. The continuous space formulation of IPP model frees the approach from of many issues that plague traditional SDM, including the size of background (Renner & Warton 2013).

Another important issue for SDMs is the need to combine multi-scale drivers in a single prediction. Data resolution influences model fit and prediction (Yates et al. 2018). Therefore, the spatial grain of predictors should match the scales over which the relevant ecological processes operate (Scales et al. 2017). Our chosen IPP model also overcomes this covariate-based limitation of current practice and models for SDM, which require a perfect matching of spatial grain among all predictors (e.g., fine-scale data are often spatially averaged through a coarsening process to match the scales of less finely resolved covariates). Instead, IPP models can work on predictor raster layers with mismatched spatial grain.

A third area of concern about SDMs involves the summary statistical measures. The AUC metric (the area under the receiver-operator curve), is an unreliable metric of model evaluation in traditional SDM (Lobo et al. 2008; Mainali et al. 2015). These concerns of AUC are irrelevant to our analysis with IPP models because we use the deviance of the point process distribution for model evaluation, which relies on a “local and proper scoring rule” (Gneiting & Raftery 2007). This means the scoring rule (discussed in the main text) is guaranteed to consistently find the model that provides the most accurate predictions or forecasts (Gneiting & Raftery 2007).

These major advantages of IPP over traditional SDM emerge from the fact that traditionally used presence-background regression models begin with augmenting the data assuming background as absences (Renner et al. 2015). Labelling the background points as “pseudoabsences” or “background points” makes no difference to the model, which treats the background points as absence points to represent a state alternate to presence.

We emphasize that our method for comparing expert opinion against IPP-based SDMs is fully applicable to other methods of constructing SDMs. This is because the input variables for quantifying expert score includes one layer of expert-drawn binary map for species range and one layer of continuous variable representing probability. This metric can be used to evaluate binary expert opinion against continuous probability or, by extension, to compare a binary representation of a process against its continuous representation. The main advantage of our metric is in comparing a binary data against continuous data, while at the same time adjusting for differences in data availability so that the scores can be compared across studies.

References Cited:

Acevedo P, Jiménez-Valverde A, Lobo JM, Real R. 2012. Delimiting the geographical background in species distribution modelling. Journal of Biogeography **39**:1383–1390.

Lobo JM, Jiménez-Valverde A, Real R. 2008. AUC: a misleading measure of the performance of predictive distribution models. Global ecology and Biogeography **17**:145–151.

Scales KL, Hazen EL, Jacox MG, Edwards CA, Boustany AM, Oliver MJ, Bograd SJ. 2017. Scale of inference: on the sensitivity of habitat models for wide-ranging marine predators to the resolution of environmental data. Ecography **40**:210–220.

Yates KL et al. 2018. Outstanding challenges in the transferability of ecological models. Trends in Ecology & Evolution **33**:790–802.

Appendix S4. (a) Dependence of Scott Expert Score to area of its own range and (b) that of Glassberg Expert Score to area of its own range.

Appendix S5. Explanatory variables of Expert Score for species with non-truncated ranges. Description of the plots as in Fig 2 in main text.

Appendix S6. Significant coefficients of Glassberg Expert Score when the response variable was predicted by each of the three groups of predictors separately. (a) Predictors related to attributes of expert maps, (b) Predictors related to attributes of occurrences, (c) Predictors related to the ecology/life history traits of butterfly. The predictors are mean-centered and scaled by 1 s.d. to show the effects clearly on the same scale of estimate. Mean and 95% confidence interval of the coefficient estimates shown.

Appendix S7. Significant coefficients of Scott Expert Score when the response variable was predicted by each of the three groups of predictors separately. (a) Predictors related to attributes of expert maps, (b) Predictors related to attributes of occurrences, (c) Predictors related to the ecology/life history traits of butterfly. The predictors are mean-centered and scaled by 1 s.d. to show the effects clearly on the same scale of estimate. Mean and 95% confidence interval of the coefficient estimates shown.

Appendix S8. Bivariate plots of Expert Agreement against its explanatory variables with significant coefficients.

Appendix S9. Significant coefficients of Expert Agreement when the response variable was predicted by each of the two groups of predictors separately. (a) Predictors related to life history traits of butterfly, (b) Predictors related to attributes of occurrences. The predictors are mean-centered and scaled by 1 s.d. to show the effects clearly on the same scale of estimate. Mean and 95% confidence interval of the coefficient estimates shown.

Appendix S10. Variance of Expert Agreement explained by the covariates related to occurrence geometry and life history for the species with non-truncated ranges.

Appendix S11. What attributes of map geometry yield reliable expert maps of a species range? Reported are best fit linear regressions between Glassberg Expert Score and attributes of map geometry, with model fit shown in parentheses. See Table 1 for definition of predictors.

*Analyses of all species (truncated and non-truncated ranges); bivariate plots shown in Fig 2c-e*

Expert Score = −0.60295 * Convex Hull Score + 0.93826 (Adj. R^2^ = 0.33)

Expert Score = −0.0023236 * Ratio of Area to Perimeter + 0.8498739 (Adj. R^2^ = 0.47)

Expert Score = −0.16304 * Polsby−Popper Score + 0.62931 (Adj. R^2^ = 0.01)

*Analyses of species without any range truncation; bivariate plots shown in Appendix S5, panels c-e*

Expert Score = −0.61113 * Convex Hull Score + 0.89946 (Adj. R^2^ = 0.46)

Expert Score = −0.0023685 * Ratio of Area to Perimeter + 0.7906476 (Adj. R^2^ = 0.47)

Expert Score = −0.3763 * Polsby−Popper Score + 0.6257 (Adj. R^2^ = 0.05)
